# Association between socio-economic factors and HIV self-testing knowledge amongst South African women

**DOI:** 10.4102/sajhivmed.v23i1.1347

**Published:** 2022-03-24

**Authors:** Michael Ekholuenetale, Chimezie I. Nzoputam, Osaretin C. Okonji

**Affiliations:** 1 Department of Epidemiology and Medical Statistics, Faculty of Public Health, College of Medicine, University of Ibadan, Ibadan, Nigeria; 2 Department of Public Health, Center of Excellence in Reproductive Health Innovation (CERHI), College of Medical Sciences, University of Benin, Benin City, Nigeria; 3 Department of Medical Biochemistry, School of Basic Medical Sciences, University of Benin, Benin City, Nigeria; 4 School of Pharmacy, Faculty of Natural Science, University of the Western Cape, Cape Town, South Africa

**Keywords:** HIV, South Africa, women, HIV prevention, self-testing, HIV testing

## Abstract

**Background:**

Self-testing for HIV is an effective and alternative method of increasing HIV testing rates and a strategy for reaching populations that are underserved by HIV testing services. Nonetheless, many resource-constrained settings are yet to adopt HIV self-testing (HIVST) into their national HIV programmes.

**Objectives:**

This study aimed to examine the association between socio-economic factors and HIVST knowledge amongst South African women.

**Method:**

We used nationally representative data from the 2016 South African Demographic and Health Survey. A sample of 8182 women of reproductive age was analysed. The outcome variable was HIVST knowledge. This was measured dichotomously; know versus do not know about HIVST. The multivariable logistic model was used to examine the measures of association, with the level of significance set at *P* < 0.05.

**Results:**

The prevalence rate of HIVST knowledge was found to be approximately 24.5% (95% confidence interval [CI]: 22.9–26.1) amongst South African women. Women with tertiary education were 3.93 times more likely to have HIVST knowledge, when compared with those with no formal education (odds ratio [OR]: 3.93; 95% CI: 1.37–11.26). Rural residents had a 33% reduction in HIVST knowledge when compared with those residing in urban areas (OR: 0.67; 95% CI: 0.51–0.89). The odds of interaction between the richer and richest women who have good knowledge of HIV infection were 1.88 and 2.24 times more likely to have HIVST knowledge, respectively, when compared with those from the poorest wealth household who have good knowledge of HIV infection.

**Conclusion:**

Based on the low level of HIVST knowledge, the findings emphasise the importance of developing effective HIVST educational campaigns. Moreover, programmes should be designed to address the unique needs of the socio-economically disadvantaged women.

## Introduction

The Southern African region appears to be the epicentre of the HIV epidemic, accounting for 54.5% of the 38 million people living with HIV (PLHIV), 43.0% of all new HIV infections and 43.0% of all AIDS-related deaths worldwide.^1,2^ Nine of the 10 countries with the highest prevalence rates of HIV worldwide are located in this region in descending order: Eswatini (Swaziland), Lesotho, Botswana, South Africa, Zimbabwe, Mozambique, Namibia, Zambia and Malawi, with prevalence rates ranging from 27.0% to 8.9%.^1,2^ The rate of new HIV infections and AIDS-related deaths dropped by 38.0% and 49.0%, respectively, in 2019 compared with 2010 figures.^2^ In 2019, nearly 60.0% of new HIV infections were reported amongst women: adolescent girls and young women between 15 years and 24 years accounted for 26.0% of the new HIV infections – a group 2.5 times more likely to become infected with HIV than their male counterparts.^2^

HIV self-testing (HIVST) is a testing approach that has the potential to complement traditional HIV counselling and testing (HCT) programmes by including hard-to-access populations in its reach.^3^ Based on the available studies, knowledge of HIVST in the general community ranges between 14.0% and 69.9%.^4,5,6,7,8^ Its awareness is found to be greater amongst those with high socio-economic status, those with tertiary education and those at an increased risk of acquiring HIV.^4,5,6,7^ Demographic and socio-economic factors influence knowledge or awareness of HIVST. This varies by age group, sex, level of education, marital status, wealth status, place of residence and geographical region, exposure to media and HIV stigma.^4,5,6,7^ Women, older individuals, those with lower educational achievement and low socio-economic status are groups who are less aware of HIVST.^4,5,6,7^

The knowledge of HIVST is reportedly low amongst men and women in South Africa, with men being more aware than women.^4,9^ A study that looked at HIV testing and self-testing coverage amongst men and women in South Africa indicated that awareness of self-testing was low (2.02%), and that a very few (2.90%) respondents had ever self-tested for HIV.^4^ The study also showed that highly educated individuals, those living in wealthy households, urban residence and those often exposed to media had a higher awareness of HIVST.^4^ Although the main source of HIVST awareness amongst women in South Africa was media channels,^9^ the lack of HIVST awareness or knowledge amongst this group may have resulted from gaps in HIVST education within primary health care facilities and deficiencies in clinical research.^9^ Additional factors include the lack of HIV counselling, fear of a positive HIV result and failure to link to care.^9^

The knowledge of HIVST is especially important amongst women in communities with high HIV burden such as South Africa, where safe sex practices are not followed and sexual concurrency is frequent.^10^ Communal knowledge of HIVST is essential for the success of prevention programmes and for the realisation of the United Nations Programme on HIV/AIDS’ (UNAIDS) 95-95-95% 2030 goals.^11^ Several studies have shown that socio-economic factors drive the transmission of HIV amongst adolescent girls and young women.^10,12^ Despite the high incidence of HIV in South Africa, particularly amongst women, and the socio-economic inequalities experienced by women, there is little understanding of the actual socio-economic contributors influencing their HIVST knowledge. The objective of this study was to examine the association between socioe-conomic factors and HIVST knowledge amongst South African women.

## Methods

### Study design

This research study was based on a cross-sectional household survey, the South African Demographic and Health Survey (SADHS) of 2016.^13^ This used a stratified two-stage sample design, with sampling probability proportional to the size of primary sampling units (PSUs) in the first stage and systematic sampling of dwelling units (DUs) in the second stage. The sampling frame used in the survey is the Statistics South Africa Master Sample Frame (MSF), which was created using Census 2011 enumeration areas (EAs). The MSF treated EAs of manageable size as PSUs. Small neighbouring EAs were pooled together to form ‘new’ PSUs and large EAs were split into conceptual PSUs. The frame includes information about each PSU’s geographic type (urban, traditional or farm) and the estimated number of residential DUs. The size of PSU was calculated using the Census 2011 DU count. A total of 750 PSUs were chosen from the 26 sampling strata, with 468 chosen from urban areas, 224 from traditional areas and 58 from farm areas.

### Data source

We used nationally representative cross-sectional data from the 2016 SADHS. A sample of 8182 women aged 15–49 years were extracted from the individual questionnaires of women. These data connect survey responses to HIV test results derived from biomarker data. The Inner City Fund provided technical assistance throughout the survey programme, with funding support from the United States Agency for International Development (USAID). The goal of the survey was to collect current and reliable data on fertility, family planning, infant and child mortality, maternal and child health, nutrition, domestic violence, and knowledge and prevalence of HIV and other non-communicable diseases, so that progress on these issues could be tracked over time. The data are publicly available and can be accessed at; http://dhsprogram.com/data/available-datasets.cfm. Details of the Demographic and Health Survey (DHS) sampling procedure have been previously reported.^13^

### Variable selection and measurement

#### Outcome

The dependent variable was based on the question ‘knowledge and use of HIV test kits’; a value of 1 or 0 indicated whether a respondent has heard of HIV test kits. Women who responded ‘has tested with HIV test kits’ or ‘knows test kits but never tested with them’ were coded as ‘1’, whilst those who responded ‘never heard of HIV test kits’ were coded ‘0’.

#### Explanatory variables

- Age (years): 15–19, 20–24, 25–29, 30–34, 35–39, 40–44, 45–49
- Region: Western Cape, Eastern Cape, Northern Cape, Free State, KwaZulu-Natal, North West, Gauteng, Mpumalanga and Limpopo
- Ethnicity: black or African people, white people, mixed race people, Indian or Asian people, and other
- Frequency of reading newspaper or magazine: not at all, less than once a week and at least once a week
- Frequency of listening to radio: not at all, less than once a week and at least once a week
- Frequency of watching television: not at all, less than once a week and at least once a week
- Marital status: single, currently married or in union, and formerly married
- Family motility: < 5 years versus long-term residency (5+ years)
- Gender of household head: male versus female
- Knowledge of HIV.

HIV-related knowledge was computed as the sum of the correct answers to vital questions. For questions assessing HIV knowledge, answers were recoded as follows: correct answer = 1, incorrect answer = 0 and do not know = 0. Twelve questions were included in the HIV infection knowledge total score, giving a highest possible total score of 12. We computed the mean value of the scores. A respondent with a score below the mean value was classified to have poor knowledge. A respondent with the score at or above the mean value was classified as having ‘good knowledge’. Table 1 lists questions on the HIV-related knowledge. The inclusion of the factors was based on previous studies.^14,15,16^

**TABLE 1:** Questions included in the computation of the HIV infection-related knowledge scores.

HIV infection knowledge question	Coding	
	Yes	No
Ever heard of AIDS	1	0
HIV transmitted during pregnancy	1	0
HIV transmitted during delivery	1	0
HIV transmitted by breastfeeding	1	0
Know a place to get HIV test	1	0
Reduce the risk of getting HIV: do not have sex at all	1	0
Reduce the risk of getting HIV: always use condoms during sex	1	0
Reduce the risk of getting HIV: have one sex partner only, who has no other partners	1	0
Can get HIV from mosquito bites	0	1
Can get HIV by sharing food with person who has AIDS	0	1
A healthy-looking person can have HIV	1	0
Can get HIV by witchcraft or supernatural means	0	1

#### Socio-economic variables

Women’s educational level, household wealth, and residential and employment status were selected as the socio-economic factors in this study. Previous studies^12,17,18,19^ also used these factors whilst investigating for socio-economic factors. Women’s education was categorised as no formal education, primary, secondary and higher. The place of residence was categorised as urban or rural. Employment status: yes, if currently employed versus no, if unemployed. The procedure to determine household wealth is complex but is elaborated in detail in a previous study by authors of this study.^12^

### Statistical analysis

To adjust for the sampling design, the survey module (‘svy’) command was used. Multicollinearity, which is known to be a major source of concern in regression models, was determined using a variance inflation factor of 10.^20^ Nevertheless, no variable was removed from the model because they were determined to be unrelated. In univariate and bivariate analyses, the percentage and chi-square tests were used, respectively.

It is assumed that respondents who are living with HIV will have greater knowledge of HIVST. We examined the interaction between HIV knowledge and socio-economic factors to confirm or reject this assumption.

The predictive marginal effect model included all significant variables from the bivariate analysis (with corresponding 95% CI). The predictive marginal effect model is presented thus as follows:


$$\mathrm{Pr}\left(Y=1|\text{Set}[\text{E}=\text{e}]\right)=\sum_{z}{\hat{P}}_{ez}\mathrm{Pr}(Z=z),$$ [Eqn 1]

where Set [E = e] reflects putting all observations to a single exposure level e and *Z* = *z* refers to a given set of observed values for the covariate vector *Z*. Furthermore,is the predicted probabilities of HIVST knowledge for any E = e and *Z* = *z*. The marginal effects indicate a weighted average over the distribution of the covariates and are equal to estimates obtained by standardising the entire population. As a post logit test, exposure *E* is set to the level *e* for all women in the data set, and the logit coefficients are used to compute predicted probabilities for every woman at their observed covariate pattern and newly exposure value. Because predicted probabilities are computed under the same distribution of *Z,* there is no covariate of the corresponding effect measure estimates.^21,22^ Statistical significance set at *P* < 0.05 (STATA version 14, StataCorp., College Station, TX, United States [US]) was used for the data analysis.

### Ethical considerations

This study is a secondary analysis of data derived from the 2016 South African Demographic and Health Survey (SADHS) and anonymised of any identifier information for this investigation. The survey protocol was reviewed and approved by the South African Medical Research Council (SAMRC) Ethics Committee and the Inner City Fund (ICF) Institutional Review Board. MEASURE DHS/ICF International granted the authors permission to use the data. The DHS programme adheres to industry norms for preserving the privacy of respondents. ICF International assures that the survey complies with the Human Subjects Protection Act of the United States Department of Health and Human Services.

## Results

The weighted prevalence of HIVST knowledge in the entire cohort surveyed was approximately 1849/8182 (24.5%; 95% CI: 22.9–26.1).

The distribution of HIVST knowledge across women in South Africa is discussed, as presented in Table 2. The prevalence rate of HIVST knowledge was found to be 25.6% amongst women with good knowledge of HIV infection (25.6%; 95% CI: 23.8–27.5). Furthermore, the distribution of HIVST knowledge was 48.9% amongst women with tertiary education (48.9%; 95% CI: 44.8–53.0), 34.8% amongst women from the richest households (34.8%; 95% CI: 31.0–38.9), 28.7% amongst women from urban residence (28.7%; 95% CI: 26.6–31.0) and 33.8% amongst women who are employed (33.8%; 95% CI: 31.1–36.6), respectively. The details of results are presented in Table 2.

**TABLE 2:** Distribution of HIV self-testing knowledge amongst South African women (*N* = 8182).

Variable	*n*	%	Prevalence of HIVST knowledge (%)	95% CI	*P*
**HIV infection knowledge**					0.008*
Poor	2568	31.4	21.8	19.5–24.2	
Good	5614	68.6	25.6	23.8–27.5	
**Education**					< 0.001*
No formal education	176	2.2	11.2	6.1–19.7	
Primary	816	10.0	11.1	8.7–14.0	
Secondary	6335	77.4	22.6	21.0–24.2	
Tertiary	855	10.4	48.9	44.8–53.0	
**Household wealth**					< 0.001*
Poorest	1530	18.7	15.0	12.3–18.1	
Poorer	1743	21.3	21.1	18.0–24.7	
Middle	1818	22.2	22.5	19.4–25.9	
Richer	1665	20.4	30.7	27.8–33.7	
Richest	1426	17.4	34.8	31.0–38.9	
**Residential status**					< 0.001*
Urban	4653	56.9	28.7	26.6–31.0	
Rural	3529	43.1	15.7	13.9–17.7	
**Employment**					< 0.001*
No	5500	67.2	19.5	17.9–21.1	
Yes	2682	32.8	33.8	31.1–36.6	
**Age (years)**					< 0.001*
15–19	1413	17.3	14.5	12.1–17.2	
20–24	1356	16.6	27.3	24.0–31.0	
25–29	1352	16.5	27.2	23.8–30.9	
30–34	1247	15.2	26.8	23.6–30.3	
35–39	1001	12.2	27.7	24.0–31.6	
40–44	929	11.4	25.4	21.5–30.0	
45–49	884	10.8	23.3	19.5–27.4	
**Region**					< 0.001*
Western Cape	626	7.7	27.7	24.3–31.5	
Eastern Cape	1024	12.5	17.0	14.4–20.1	
Northern Cape	688	8.4	23.5	19.8–27.7	
Free State	839	10.3	28.7	25.4–32.3	
KwaZulu-Natal	1273	15.6	21.2	17.8–25.0	
North West	852	10.4	26.0	21.9–30.5	
Gauteng	807	9.9	31.3	26.8–36.1	
Mpumalanga	1048	12.8	20.5	17.6–23.7	
Limpopo	1025	12.5	17.4	14.6–20.7	
**Ethnicity**					< 0.001*
Black or African people	7070	86.4	23.7	22.0–25.4	
White people	200	2.4	36.5	29.1–44.5	
Mixed race people	821	10.0	26.6	23.2–30.3	
Indian or Asian people	88	1.1	35.8	25.6–47.5	
Other	3	0.1	42.8	5.1–91.3	
**Frequency of reading newspaper or magazine**					< 0.001*
Not at all	3074	37.6	14.1	12.5–15.9	
Less than once a week	2161	26.4	23.7	21.2–26.3	
At least once a week	2947	36.0	34.0	31.6–36.5	
**Frequency of listening to radio**					< 0.001*
Not at all	2517	30.8	15.1	13.3–17.0	
Less than once a week	1330	16.3	23.3	20.1–26.9	
At least once a week	4335	53.0	29.8	27.9–31.9	
**Frequency of watching television**					< 0.001*
Not at all	1444	17.7	11.9	9.8–14.3	
Less than once a week	778	9.5	25.0	21.4–28.9	
At least once a week	5960	72.8	27.3	25.5–29.2	
**Marital status**					0.003*
Never in union	4905	60.0	22.6	20.9–24.5	
Currently in union or living with a man	2752	33.6	27.4	24.8–30.1	
Formerly in union	525	6.4	25.2	20.4–30.8	
**Family mobility**					0.024*
< 5 years	1720	21.0	27.5	24.1–31.3	
Long-term residency (5+ years)	6462	79.0	23.6	22.1–25.2	
**Gender of household head**					0.072
Male	3470	42.4	25.9	23.6–28.5	
Female	4712	57.6	23.3	21.6–25.2	

CI, confidence interval; HIVST, HIV self-testing.

* , Significant at *P* < 0.05.

In Table 3, we present the factors associated with HIVST knowledge amongst South African women. Women with tertiary education were 3.93 times more likely to have HIVST knowledge when compared with those with no formal education (odds ratio [OR]: 3.93; 95% CI: 1.37–11.26). Rural residents had a 33% reduction in HIVST knowledge when compared with those residing in urban areas (OR: 0.67; 95% CI: 0.51–0.89). The odds of an interaction between the richer and richest women who have good knowledge of HIV infection were 1.88 and 2.24 times more likely to have HIVST knowledge, respectively, than those from the poorest wealth household who have good knowledge of HIV infection. In addition, women aged 20–24, 25–25, 30–34 and 35–39 years were 1.81, 1.55, 1.46 and 1.52 times more likely to have good HIVST knowledge, respectively, when compared with those aged 15–19 years (see details in Table 3).

**TABLE 3:** Measures of the association of factors linked to HIV self-testing knowledge amongst South African women.

Variable	aOR	95% CI	*P*
**HIV infection knowledge**			
Poor	1.00	-	-
Good	0.81	0.20–3.35	0.773
**Education**			
No formal education	1.00	-	-
Primary	1.04	0.36–3.00	0.937
Secondary	1.47	0.55–3.94	0.448
Tertiary	3.93	1.37–11.26	0.011*
**Education # HIV infection knowledge**			
No formal education # good	1.00	-	-
Primary # good	0.83	0.20–3.43	0.801
Secondary # good	0.87	0.24–3.11	0.826
Tertiary # good	0.60	0.16–2.26	0.446
**Household wealth**			
Poorest	1.00	-	-
Poorer	0.97	0.63–1.50	0.906
Middle	1.38	0.89–2.14	0.154
Richer	1.14	0.72–1.81	0.577
Richest	1.04	0.62–1.72	0.891
**Household wealth # HIV infection knowledge**			
Poorest # good	1.00	-	-
Poorer # good	1.49	0.85–2.63	0.166
Middle # good	1.03	0.59–1.78	0.915
Richer # good	1.88	1.05–3.39	0.035*
Richest # good	2.24	1.24–4.07	0.008*
**Residential status**			
Urban	1.00	-	-
Rural	0.67	0.51–0.89	0.005*
**Residential status # HIV infection knowledge**			
Urban # good	1.00	-	-
Rural # good	0.84	0.63–1.13	0.256
**Employment**			
No	1.00	-	-
Yes	1.27	0.95–1.72	0.111
**Employment # HIV infection knowledge**			
No # good	1.00	-	-
Yes # good	1.28	0.91–1.81	0.161
**Age (years)**			
15–19	1.00	-	-
20–24	1.81	1.39–2.35	< 0.001*
25–29	1.55	1.17–2.06	0.002*
30–34	1.46	1.09–1.95	0.010*
35–39	1.52	1.14–2.04	0.005*
40–44	1.30	0.95–1.76	0.098
45–49	1.31	0.94–1.82	0.114
**Region**			
Western Cape	1.00	-	-
Eastern Cape	1.07	0.79–1.47	0.650
Northern Cape	1.26	0.95–1.66	0.105
Free State	1.41	1.05–1.91	0.023*
KwaZulu-Natal	1.08	0.77–1.52	0.638
North West	1.56	1.13–2.15	0.006*
Gauteng	1.40	1.01–1.93	0.042*
Mpumalanga	1.23	0.90–1.68	0.200
Limpopo	1.17	0.82–1.68	0.384
**Ethnicity**			
Black or African people	1.00	-	-
White people	0.73	0.49–1.10	0.124
Mixed race people	0.86	0.65–1.15	0.310
Indian or Asian people	1.03	0.58–1.84	0.914
Other	0.92	0.06–14.23	0.952
**Frequency of reading newspaper/magazine**			
Not at all	1.00	-	-
Less than once a week	1.46	1.22–1.75	<0.001*
At least once a week	1.84	1.51–2.24	<0.001*
**Frequency of listening to radio**			
Not at all	1.00	-	-
Less than once a week	1.23	0.98–1.53	0.069
At least once a week	1.41	1.18–1.69	<0.001*
**Frequency of watching television**			
Not at all	1.00	-	-
Less than once a week	1.43	1.03–1.99	0.033*
At least once a week	1.21	0.91–1.61	0.198
**Marital status**			
Never in union	1.00	-	-
Currently in union or living with a man	1.03	0.87–1.22	0.764
Formerly in union	1.11	0.80–1.53	0.530
**Family mobility**			
< 5 years	1.00	-	-
Long-term residency (5+ years)	0.80	0.66–0.96	0.016*

CI, confidence interval; aOR, adjusted odds ratio.

* , Significant at *P* < 0.05.

Table 1-A1 (Appendix 1) shows the predictive marginal interaction effect of HIVST knowledge by socio-economic factors. The marginal predictive analysis was conducted to decipher the effects of socio-economic factors on HIVST knowledge whilst adjusting for women’s characteristics. From the results of the predictive marginal effects, assuming that the distribution of all factors remained the same, but every woman had secondary or tertiary education, we would expect 23.0% or 36.6% of HIVST knowledge, respectively. If every woman had secondary education and good HIV general knowledge or had tertiary education and good HIV general knowledge, we would expect 23.5% or 34.8% of HIVST knowledge, respectively. Interactively, if every woman was in the richest household and had good HIV general knowledge, we would expect 31.0% of HIVST knowledge. Moreover, if every woman was an urban dweller or if every woman was employed, we would expect 26.9% or 28.8% of HIVST knowledge, respectively. We found an increased marginal interaction effect between the urban dwellers and those having good general knowledge of HIV, and being employed and having good general knowledge of HIV than their counterparts, respectively. The details of the predictive marginal interaction effects of HIVST knowledge by socio-economic factors are shown in Table 1-A1.

**FIGURE 1:**
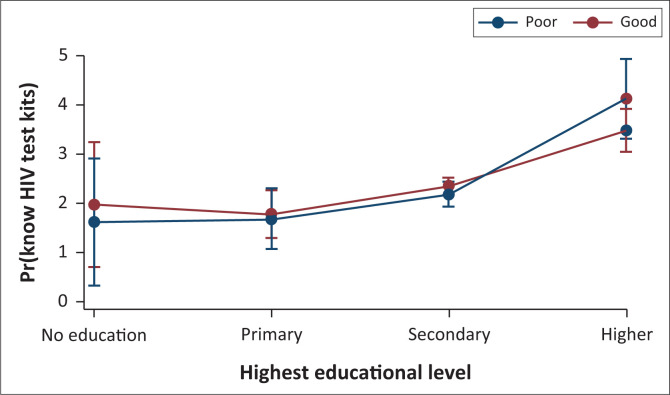
Predictive marginal effects of HIV test kits knowledge by educational level and HIV infection knowledge.

As shown in Figure 1, the marginal effects plot of HIVST knowledge by educational attainment and HIV infection knowledge is presented. The marginal interaction effects of HIVST knowledge were greater for women who had tertiary education than those with no formal education who had good HIV infection knowledge.

**FIGURE 2:**
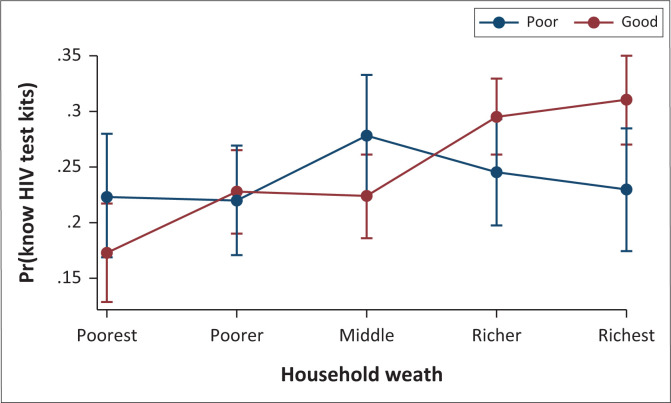
Predictive marginal effects of HIV test kits knowledge by household wealth and HIV infection knowledge.

As presented in Figure 2, based on the marginal effects plot of HIVST knowledge by household wealth and HIV infection knowledge, the marginal interaction effects of HIVST knowledge were higher amongst women having good knowledge of HIV infection (brown line), particularly in the richer and richest households.

**FIGURE 3:**
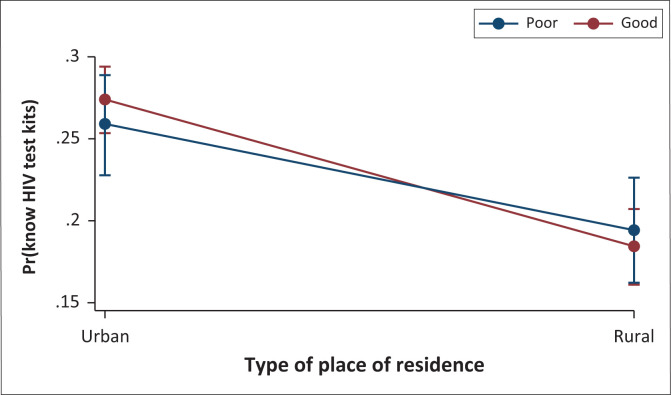
Predictive marginal effects of HIV test kits knowledge by residential status and HIV infection knowledge.

As shown in Figure 3, according to the marginal effects plot of HIVST knowledge by residential status and HIV infection knowledge, the marginal interaction effects of HIVST knowledge were found to be higher amongst women in the urban residence.

**FIGURE 4:**
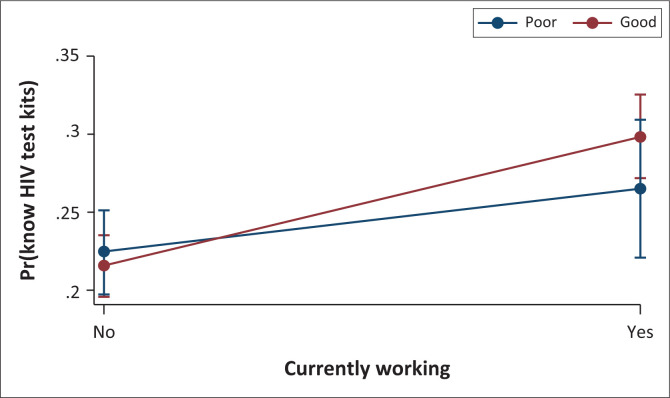
Predictive marginal effects of HIV test kits knowledge by employment status and HIV infection knowledge.

Figure 4 presents the marginal effects plot of HIVST knowledge by employment status and HIV infection knowledge. The marginal interaction effects of HIVST knowledge were higher amongst women who are employed and have good knowledge of HIV infection.

## Discussion

We examined the knowledge of HIVST amongst South African women using a nationally representative large data set. The prevalence rate of HIVST knowledge in this group was approximately 24.5%, which was higher than that reported amongst the Malawian (11.4%) and Zimbabwean (14.5%) population, respectively.^23^ More effort is needed to implement evidence-based HIVST interventions to reach women, to both improve their knowledge and practice, such as through healthcare facilities and antenatal care in high HIV-burden settings, or through networks of other high-risk sexual and social contacts, including those with HIV.^24^

Fear of discovering one’s HIV status may be behind the lack of knowledge or awareness of self-testing.^25,26,27^ Social marketing improves the knowledge or uptake of HIVST^28^ as observed in studies involving men who have sex with men (MSM).^29,30,31^ In this study, exposure to mass media was positively associated with HIVST knowledge. Social marketing of key messages and strategies that promote HIVST on mass media platforms are likely to be impactful in Africa.^25,32,33^

In this study educated, compared with uneducated, women, had a greater knowledge of HIVST. Furthermore, the knowledge of HIVST amongst women was found to increase with educational advance from primary to tertiary levels, the outcome of which has been shown in other studies.^26,34,35^ Education assists with knowing one’s HIV status: lower levels of education correlate with less knowledge of HIV infection and a lower uptake of HIV services.^12,35,36,37^

Wealth is correlated with greater knowledge of HIVST. Better HIVST knowledge was observed amongst employed women than unemployed. Although these findings have been reported inconsistently,^38,39^ employment brings financial independence and independence with regard to health decisions. When women are denied such freedoms, their health, including HIV self-knowledge, may be compromised. In order to mitigate this challenge, community sensitisation, social mobilisation and women’s empowerment should be considered a key intervention in women’s HIVST. Wealth is correlated with improved knowledge of HIVST by facilitating access to health information, facilities and choices, and providing access to people in the know.^40^

Where you live matters. Rural people are more likely to be underserved with healthcare services and to experience barriers in access to health information.^41,42^ Knowledge of HIV in this study varied by place of residence. Lack of access to appropriate health information could be improved by better media coverage of health issues. Nevertheless, media reporting on health issues is of varying quality, particularly messages about HIV testing, counselling and treatments.^43,44^ During the coronavirus disease 2019 (COVID-19) pandemic, the media has played a very important role in supporting all citizens to make informed choices. Why can this not be performed with regard to HIVST too?

There is an increased HIVST knowledge interaction effect by high socio-economic status and good HIV infection knowledge. Increase in wealth was observed to have a positive marginal interaction effect with increased HIV infection knowledge amongst the South African women. As the wealth status of the women increased, their knowledge of HIVST also increased. This implies that having good knowledge of HIV infection and being from a wealthy household are associated with having good knowledge of HIVST amongst the study population. Hence, it is necessary to promote women’s health, particularly their sexual and reproductive health, and ways in which communities can engage in advancing the rights of women to make informed health decisions. The fact that this study’s results revealed that poor and illiterate women had lower levels of HIVST knowledge validates the existing global perspectives on the association between low socio-economic status and poor health outcomes, such as HIV infection.^45,46^ As a result of their low socio-economic status, the poor or under privileged and those with low educational attainment could face the dual problem of high vulnerability and a lack of opportunities to make better health choices (such as access to information on prevention, testing and counselling for HIV infection). Besides the socio-economic status, other contextual factors associated with low knowledge of HIVST should be identified and addressed simultaneously.

It is crucial to undertake interventions that incorporate specific designs targeted at women of low socio-economic status. Women’s empowerment, decision-making authority, girl-child education and women’s autonomy, for example, could favourably influence the utilisation of healthcare services, including HIV prevention, in South Africa. The government, non-governmental organisations and other stakeholders in the healthcare system should create and promote key interventions, such as free HIV screening or testing, as well as counselling and treatment for HIV-positive women.^47^ This will encourage more women, particularly those from poor backgrounds, to participate in HIV prevention, control and treatment programmes. Furthermore, the government and support groups will be required to enrol low-income, HIV-positive women in a specific financial assistance programme.^48^ Such a strategy might be aimed at providing economic assistance to underprivileged women, as well as lowering their HIV burden. Furthermore, special messaging aimed at increasing awareness and education of HIV amongst low-income women, the uneducated, or those living in difficult-to-reach regions might be beneficial in the battle against HIV. Women aged 20–39 years, those from Free State, North West and Gauteng were more likely to have good knowledge of HIVST when compared with those aged 15–19 years and those from Western Cape. However, women who were long-term residents were less likely to have good knowledge of HIVST when compared with those who lived in the household less than 5 years. This is consistent with previous findings that demographic characteristics were associated with HIVST knowledge.^4^

### Strengths and limitations

The strengths and limitations of this study are similar to those reported in a previous study, which used DHS data.^12^ For example, this study used nationally representative data, which is suitable for making plausible comparisons. However, data from a cross-sectional study were analysed, and therefore only association and not causality can be determined. Another limitation is the assumption that respondents who are living with HIV will have a greater knowledge of HIVST.

## Conclusion

According to the findings of this study, the knowledge of HIVST is relatively low amongst South African women. In addition, socio-economic factors were associated with HIVST knowledge. This study has a wide range of implications. Because of the low level of HIVST knowledge amongst women of reproductive age, the findings emphasise the importance of developing effective HIVST educational campaigns. It is clear that low socio-economic status was associated with a low level of HIVST knowledge, and, hence, programmes should be designed to address the unique needs of disadvantaged individuals. Furthermore, when developing educational campaigns, it is essential to consider the interaction impact of socio-economic status and HIV infection knowledge associated with HIVST. Even though local facilities can now sell HIVST kits, purchase and uptake of local facility-based kits may be limited because of the relatively low level of HIVST knowledge. As a result, it is important to not only raise awareness about the existence of HIVST but also provide information about where and how to obtain test kits, and how to use them.

## Appendix 1

**TABLE 1-A1:** Marginal interaction effect of HIV self-testing knowledge amongst South African women.

Variable	Marginal effect	95% CI	*P*
**HIV infection knowledge**			
Poor	24.0	21.6–26.4	< 0.001*
Good	24.8	23.2–26.3	< 0.001*
**Education**			
No formal education	18.8	8.8–28.8	< 0.001*
Primary	17.5	13.7–21.3	< 0.001*
Secondary	23.0	21.6–24.4	< 0.001*
Tertiary	36.6	32.6–40.5	< 0.001*
**Education # HIV infection knowledge**			
No formal education # poor	16.2	3.4–29.0	0.013*
No formal education # good	19.8	7.1–32.4	0.002*
Primary # poor	16.8	10.8–22.7	< 0.001*
Primary # good	17.8	13.0–22.6	< 0.001*
Secondary # poor	21.8	19.3–24.3	< 0.001*
Secondary # good	23.5	21.8–25.2	< 0.001*
Tertiary # poor	41.3	33.2–49.3	< 0.001*
Higher # good	34.8	30.5–39.0	< 0.001*
**Household wealth**			
Poorest	18.7	14.9–22.5	< 0.001*
Poorer	22.6	19.5–25.7	< 0.001*
Middle	23.9	20.6–27.2	< 0.001*
Richer	28.2	25.5–30.9	< 0.001*
Richest	28.9	25.5–32.3	< 0.001*
**Household wealth # HIV infection knowledge**			
Poorest # poor	22.4	16.9–27.9	< 0.001*
Poorest # good	17.3	12.9–21.7	< 0.001*
Poorer # poor	22.0	17.1–26.9	< 0.001*
Poorer # good	22.8	19.0–26.5	< 0.001*
Middle # poor	27.8	22.4–33.3	< 0.001*
Middle # good	22.4	18.6–26.1	< 0.001*
Richer # poor	24.5	19.8–29.3	< 0.001*
Richer # good	29.5	26.1–32.9	< 0.001*
Richest # poor	23.0	17.5–28.5	< 0.001*
Richest # good	31.0	27.1–35.0	< 0.001*
**Residential status**			
Urban	26.9	25.2–28.5	< 0.001*
Rural	18.6	16.4–20.8	< 0.001*
**Residential status # HIV infection knowledge**			
Urban # poor	25.8	22.8–28.9	< 0.001*
Urban # good	27.4	25.4–29.4	< 0.001*
Rural # poor	19.4	16.2–22.7	< 0.001*
Rural # good	18.4	16.1–20.7	< 0.001*
**Employment**			
No	21.7	20.0–23.3	< 0.001*
Yes	28.8	26.4–31.1	< 0.001*
**Employment # HIV infection knowledge**			
No # poor	22.4	19.7–25.1	< 0.001*
No # good	21.5	19.6–23.5	< 0.001*
Yes # poor	26.5	22.1–30.9	< 0.001*
Yes # good	29.8	27.2–32.5	< 0.001*
**Age (years)**			
15–19	19.1	15.8–22.3	< 0.001*
20–24	28.6	25.4–31.7	< 0.001*
25–29	25.9	22.7–29.1	< 0.001*
30–34	24.8	22.1–27.6	< 0.001*
35–39	25.6	22.2–29.0	< 0.001*
40–44	22.9	19.4–26.4	< 0.001*
45–49	23.1	19.5–26.6	< 0.001*
**Region**			
Western Cape	21.3	17.9–24.7	< 0.001*
Eastern Cape	22.4	19.1–25.7	< 0.001*
Northern Cape	24.9	21.2–28.6	< 0.001*
Free State	26.9	23.8–30.0	< 0.001*
KwaZulu-Natal	22.5	19.1–25.9	< 0.001*
North West	28.6	25.0–32.3	< 0.001*
Gauteng	26.7	22.9–30.5	< 0.001*
Mpumalanga	24.5	21.5–27.5	< 0.001*
Limpopo	23.8	19.9–27.6	< 0.001*
**Ethnicity**			
Black or African people	24.9	23.5–26.4	< 0.001*
White people	20.0	14.3–25.7	< 0.001*
Mixed race people	22.6	18.3–26.8	< 0.001*
Indian or Asian people	25.4	15.9–35.0	< 0.001*
Other	23.6	−19.9–67.0	< 0.001*
**Frequency of reading newspaper or magazine**			
Not at all	18.5	16.4–20.6	< 0.001*
Less than once a week	24.3	21.8–26.8	< 0.001*
At least once a week	28.4	26.2–30.6	< 0.001*
**Frequency of listening to radio**			
Not at all	20.6	18.3–23.0	< 0.001*
Less than once a week	23.8	20.4–27.2	< 0.001*
At least once a week	26.2	24.6–27.8	< 0.001*
**Frequency of watching television**			
Not at all	21.6	17.7–25.4	< 0.001*
Less than once a week	27.4	23.5–31.4	< 0.001*
At least once a week	24.6	22.9–26.2	< 0.001*
**Marital status**			
Never in union	24.2	22.4–26.1	< 0.001*
Currently in union or living with a man	24.7	22.6–26.7	< 0.001*
Formerly in union	26.0	21.0–30.9	< 0.001*
**Family mobility**			
<5 years	27.4	24.4–30.4	< 0.001*
Long-term residency (5+ years)	23.6	22.2–25.0	< 0.001*

CI, confidence interval.

* , Significant at *P* < 0.05.
